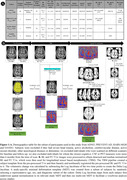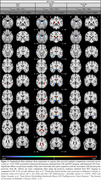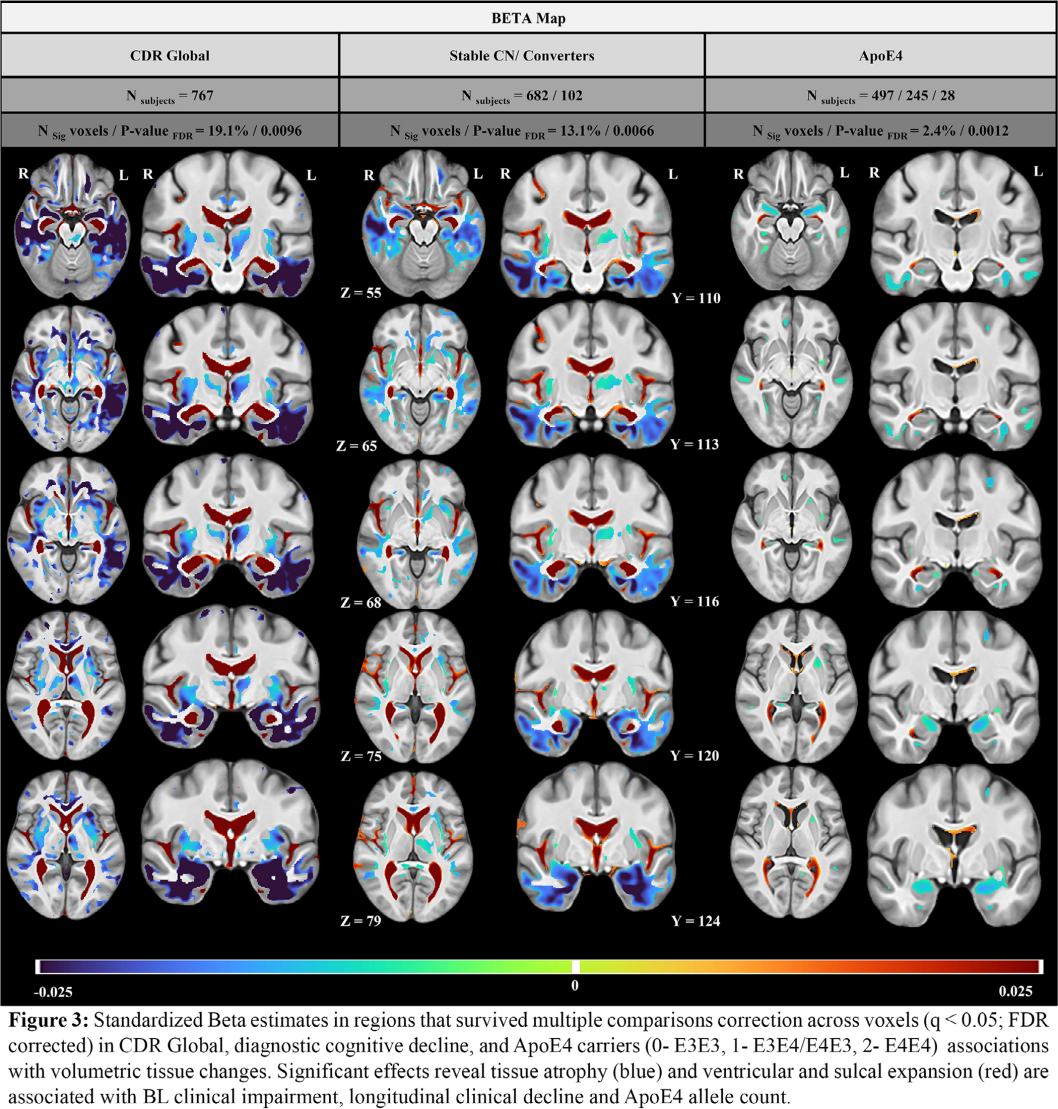# Baseline Aβ and tau markers contribute to localized patterns of brain tissue atrophy across 1223 individuals from four harmonized longitudinal datasets

**DOI:** 10.1002/alz.093008

**Published:** 2025-01-09

**Authors:** Sunanda Somu, Alyssa H Zhu, Siddharth Narula, Iyad Ba Gari, Shruti Gadewar, Talia M Nir, Neda Jahanshad

**Affiliations:** ^1^ Imaging Genetics Center, Mark and Mary Stevens Neuroimaging & Informatics Institute, University of Southern California, Marina del Rey, CA USA

## Abstract

**Background:**

Amyloid‐β (Aβ) plaques and tau pathogenesis in the brain precede cognitive decline in the progression of Alzheimer’s dementia, yet the extent to which these measures can predict localized brain tissue atrophy has not been studied in a large, diverse population. Multisite studies offer robust statistical power with larger sample sizes but are confounded by variations in biomarker quantification across studies, including variations in MRI scanners, PET tracers, and CSF assays. Longitudinal data from N=1223 individuals from four independent AD studies were harmonized to assess localized brain tissue atrophy over 2 to 5 years. We tested for associations between volumetric changes and baseline measures of Aβ, phosphorylated tau (p‐tau), and cognitive function. Associations between atrophy and ApoE4 and diagnostic conversion were also assessed.

**Method:**

Baseline (BL) and follow‐up (FU; mean=2.3+/‐0.5 years) 3T T1‐weighted brain MRI (T1w) from ADNI3 (N=373), Prevent‐AD (N=213), HABS‐MGH (N=213), and OASIS3 (N=424) were used along with corresponding PET measures obtained from FBB, FBP or PiB tracers, and CSF measures (Figure 1A). Subject‐wise maps of volumetric brain changes were created and warped to a common template for statistical analyses (Figure 1B). Voxel‐wise mixed effects linear regressions were run to test for associations between change in brain volume and Aβ‐42 positivity (CSF / PET), CSF p‐tau‐181 positivity, ApoE4 genotype, Clinical Dementia Rating (CDR), and diagnostic conversion (i.e., cognitively normal vs decline). Fixed effects included BL age, sex, scan time interval, and PET imaging tracer type or CSF processing batch; scan site was a random effect. FDR was used for voxel‐wise multiple comparisons correction.

**Result:**

Aβ and p‐tau positivity at baseline, and the ApoE4 genotype, were associated with accelerated atrophy in the temporal lobe, medial‐parietal lobe and amygdala (Figure 2). CDR and conversion were associated with ventricular expansion and atrophy in the insula, temporal lobe, hippocampus and amygdala (Figure 3).

**Conclusion:**

Our multi‐study brain mapping and harmonization approaches showed how baseline PET and CSF based amyloid and tau metrics are predictive of common and localized brain atrophy across multiple studies with diverse data collection protocols and study designs.